# A conceptual framework of cognitive-affective theory of mind: towards a precision identification of mental disorders

**DOI:** 10.1038/s44184-023-00031-0

**Published:** 2023-08-10

**Authors:** Peng Zhou, Huimin Ma, Bochao Zou, Xiaowen Zhang, Shuyan Zhao, Yuxin Lin, Yidong Wang, Lei Feng, Gang Wang

**Affiliations:** 1 School of International Studies, Zhejiang University, Hangzhou, China; 2 School of Computer and Communication Engineering, University of Science and Technology Beijing, Beijing, China; 3 Department of Foreign Languages and Literatures, Tsinghua University, Beijing, China; 4 Department of Electronic Engineering, Tsinghua University, Beijing, China; 5 National Clinical Research Center for Mental Disorders & Beijing Key Laboratory of Mental Disorders, Beijing Anding Hospital, Capital Medical University, Beijing, China; 6 Advanced Innovation Center for Human Brain Protection, Capital Medical University, Beijing, China

**Keywords:** Human behaviour, Diagnostic markers, Psychiatric disorders

## Abstract

To explore the minds of others, which is traditionally referred to as Theory of Mind (ToM), is perhaps the most fundamental ability of humans as social beings. Impairments in ToM could lead to difficulties or even deficits in social interaction. The present study focuses on two core components of ToM, the ability to infer others’ beliefs and the ability to infer others’ emotions, which we refer to as cognitive and affective ToM respectively. Charting both typical and atypical trajectories underlying the cognitive-affective ToM promises to shed light on the precision identification of mental disorders, such as depressive disorders (DD) and autism spectrum disorder (ASD). However, most prior studies failed to capture the underlying processes involved in the cognitive-affective ToM in a fine-grained manner. To address this problem, we propose an innovative conceptual framework, referred to as visual theory of mind (V-ToM), by constructing visual scenes with emotional and cognitive meanings and by depicting explicitly a four-stage process of how humans make inferences about the beliefs and emotions of others. Through recording individuals’ eye movements while looking at the visual scenes, our model enables us to accurately measure each stage involved in the computation of cognitive-affective ToM, thereby allowing us to infer about potential difficulties that might occur in each stage. Our model is based on a large sample size (*n* > 700) and a novel audio-visual paradigm using visual scenes containing cognitive-emotional meanings. Here we report the obtained differential features among healthy controls, DD and ASD individuals that overcome the subjectivity of conventional questionnaire-based assessment, and therefore could serve as valuable references for mental health applications based on AI-aided digital medicine.

## Introduction

Humans are by nature social beings. To explore the minds of others is perhaps the most fundamental ability of humans as social beings, commonly referred to as Theory of Mind (ToM)^1,2^, which begins at birth and could extend into the whole lifetime. Impairments in ToM could lead to difficulties or even deficits in social interaction. The study focuses on two disorders, depressive disorders (DD) and autism spectrum disorder (ASD), in which social impairments are commonly identified. According to the Diagnostic and Statistical Manual of Mental Disorders, fifth edition (APA, 2022), individuals with DD persistently suffer from depressed mood, markedly diminished interest or pleasure in almost all activities, feelings of worthlessness, fatigue or loss of energy and diminished ability to concentrate; individuals with ASD exhibit two clusters of symptoms: persistent deficits in social communication and social interaction, and restricted, repetitive patterns of behavior, interests, or activities. Both disorders severely impair individuals’ development, including their academic and career development as well as their overall quality of life.

ToM is often described as the ability to attribute mental states to oneself and others. It has traditionally been investigated using false-belief tasks^1,3–5^. It is generally acknowledged that typically developing (TD) children by 4 years of age can pass this explicit false-belief task, indicating the acquisition of representational ToM^6^. Note that in the standard false-belief tasks, the participants are asked to provide explicit verbal responses, which might pose particular difficulties for younger TD children and children with ASD. To solve this problem, non-verbal tasks that employ on-line techniques like eye tracking have been used to examine children’s spontaneous/implicit ToM. In a typical implicit ToM task proposed by Southgate et al.^7^, children were presented with an event where a girl was watching an object being hidden in a box; the object was then displaced while the girl was looking away. Children’s eye movements were recorded and analyzed to see whether they spontaneously anticipated the girl’s subsequent behavior on the basis of her false belief of the location of the object. The results of non-verbal ToM tasks revealed that TD children younger than 4, or even 7-month infants, already exhibited spontaneous behavioral patterns that reflected their ability to reason about others’ false beliefs^7–10^. By contrast, individuals with ASD have been found to exhibit marked difficulties in both explicit and implicit false-belief tasks. In addition, it has also been reported that high-functioning individuals with ASD who could generally pass the explicit false-belief tasks still failed in the non-verbal tasks to exhibit behavioral patterns that reflect spontaneous false-belief attribution^11–13^. In addition, research has shown that individuals with DD exhibit a significant slowdown of ToM as compared with healthy controls in false-belief tasks. (see, e.g., ref. ^14^)

In addition to the ability to reason about others’ beliefs and intentions, ToM also includes the capacity to reason about others’ emotions^15^. We refer to these two components as cognitive ToM and affective ToM respectively. The division of cognitive and affective ToM has been empirically supported by prior research. For example, Raimo and colleagues found that rather than a general decline in ToM, elder adults showed a selective decline in cognitive ToM as compared with affective ToM (e.g., ref. ^16^). Research has also shown that the two components of ToM involve brain circuitry in frontal and temporal lobes^17^. However, we wish to note that the affective ToM has been relatively understudied. Emotions are extracted from people’s appraisals of events and are often used as important indicators of their immediate reactions to events. Therefore, our sensitivity to others’ emotions, the affective ToM, plays a central role in our social life^18–20^. A meta-analysis suggests that people with DD exhibited significant deficits in affective ToM^14,21^. Previous studies also found that individuals with ASD exhibited deficits in affective ToM, which was suggested to be closely relevant to their social impairments (for a review, see ref. ^22^).

**Fig. 1:**
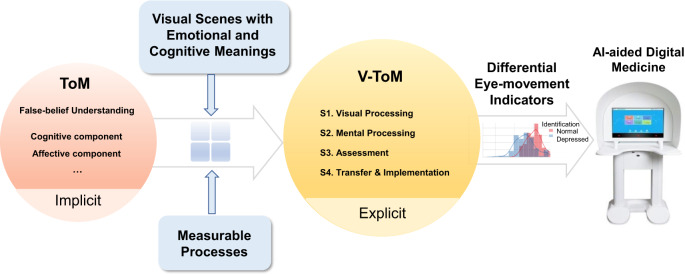
Differences between traditional ToM and our V-ToM. First, V-ToM successfully operationalizes ToM into fine-grained framework, which is realized by constructing visual scenes with emotional and cognitive meanings and by depicting explicitly a four-stage process of how humans make inferences about the beliefs and emotions of others. Second, through recording individuals’ eye movements while looking at the visual scenes, our model enables us to explicitly and accurately measure each stage involved in the computation of cognitive-affective ToM, thereby allowing us to infer about potential difficulties that might occur in each stage. The obtained differential features among normal people, DD and ASD individuals overcome the subjectivity of conventional questionnaire-based assessment, and therefore could serve as valuable references for mental health applications based on AI-aided digital medicine.

The studies reviewed so far suggest that a good model that attempts to represent ToM must take into consideration both the cognitive and affective components. However, most prior studies failed to capture the underlying processes involved in the cognitive-affective ToM in a fine-grained manner. To address this problem, we propose an innovative conceptual framework that incorporates both cognitive and affective ToM, referred to as visual theory of mind (V-ToM), by constructing visual scenes with emotional and cognitive meanings and by depicting explicitly a four-stage process of how humans make inferences about the beliefs and emotions of others (see Fig. 1). The Differences between traditional ToM and our V-ToM are as follows: First, V-ToM successfully operationalizes ToM into fine-grained framework, which is realized by constructing visual scenes with emotional and cognitive meanings and by depicting explicitly a four-stage process of how humans make inferences about the beliefs and emotions of others. Second, through recording individuals’ eye movements while looking at the visual scenes, our model enables us to explicitly and accurately measure each stage involved in the computation of cognitive-affective ToM, thereby allowing us to infer about potential difficulties that might occur in each stage. The obtained differential features among healthy controls, DD and ASD individuals overcome the subjectivity of conventional questionnaire-based assessment, and therefore could serve as valuable references for mental health applications based on AI-aided digital medicine.

More specifically, by dividing the human computational processes underlying their cognitive-affective ToM into four stages (see the middle panel of Fig. 1), our model allows us to explain how humans process social information in a more fine-grained manner. The details of each of the four stages are provided in the next section. Although the underlying processes of human cognition unfold in a dynamic and rapid manner in real time, our model makes a bold attempt to characterize these processes by dividing them into four sub-components, each with measurable features. Our model is based on traditional visual attention paradigms^23–40^, but unlike the traditional paradigms, our paradigm allows us to implement artificial intelligence (AI) algorithms when analyzing eye movement data, thereby enabling us to make inferences about potential difficulties that might occur in each of the four stages. This has important implications for understanding atypical cognitive-affective ToM like DD and ASD. By identifying the potential sources of their difficulties, we will be able to better understand the nature of their impairments, which will then lead to more effective identification and intervention programs for individuals with DD and those with ASD.

In this paper, we propose an innovative conceptual framework, V-ToM, by constructing visual scenes with emotional and cognitive meanings and by depicting explicitly a four-stage process of how humans make inferences about the beliefs and emotions of others. Although our tasks are based on individuals with DD and those with ASD, our model has the potential to be extended to other domains of ToM by incorporating new available data. In the following sections, we first introduce our conceptual framework (i.e., the V-ToM model) and then report findings from two tasks that investigated affective ToM and cognitive ToM respectively.

## Methods

### Conceptual framework

**Fig. 2:**
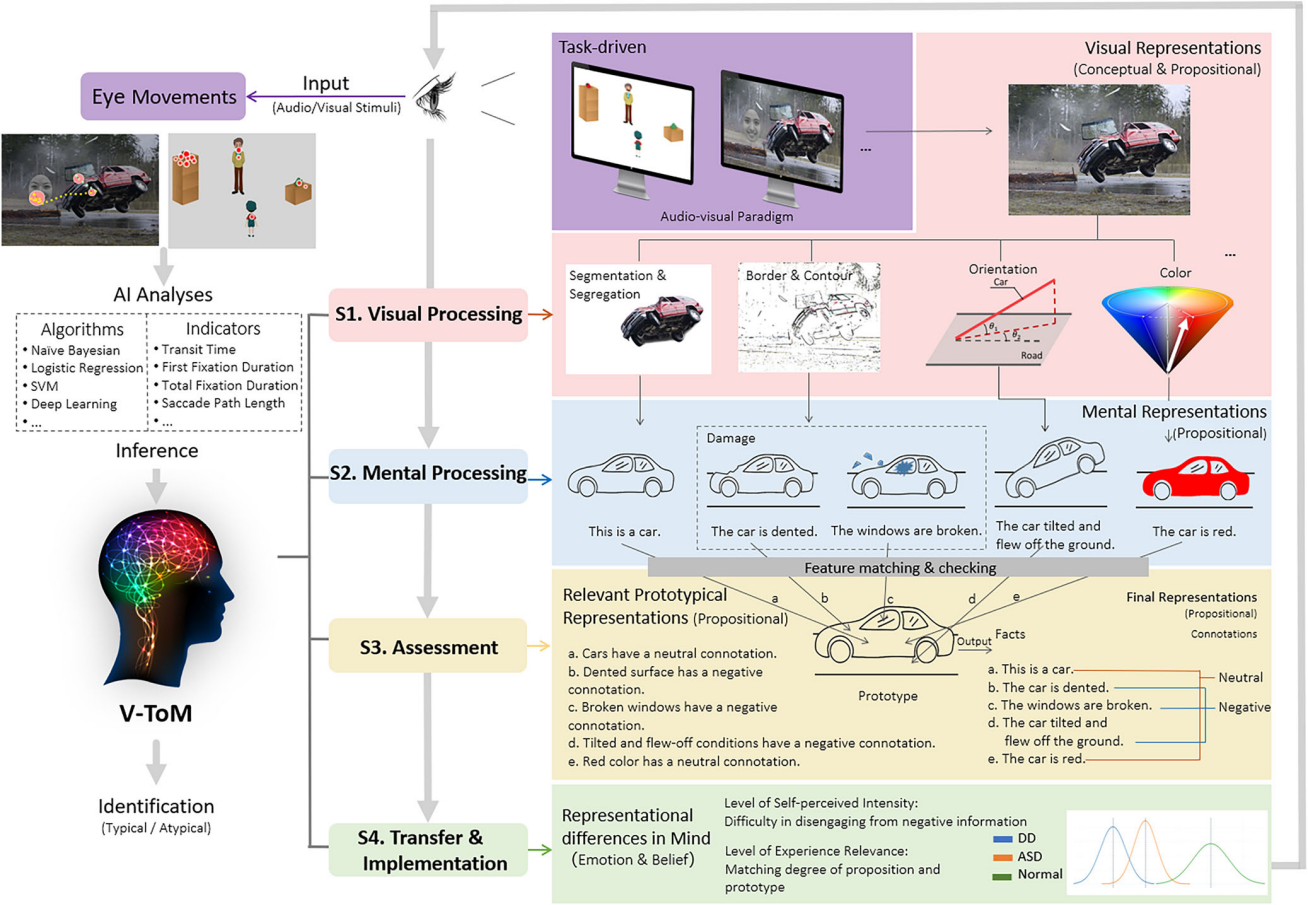
A conceptual framework of cognitive-affective ToM. The relevant cognitive processes are explicitly divided into four stages: S1. Visual processing, S2. Mental processing, S3. Assessment, and S4. Transfer and implementation. The detailed representations of each stage are provided in the color-matched boxes in the right panel.

Our model provides an account for how cognitive-affective processes can be mapped onto eye movement patterns in an established visual world, thereby allowing us to infer the underlying mental states by analyzing these eye movement patterns. As shown in Fig. 2, we divide the human cognitive processes underlying their cognitive-affective process into four stages. The detailed representations of each stage are provided in the color-matched boxes in the right panel of Fig. 2. The first stage occurs at the perceptual level that involves the processing of visual information in an established visual world. Following the feature integration theory^41^, basic features (e.g., color, orientation) are registered early and automatically at the pre-attentive stage. Thus, the first stage includes early visual processing, like the detection of basic features, the detection of border and contour, and the segmentation and segregation of figure from background (see the pink panel in Fig. 2). Visual representations that contain both conceptual and propositional properties of the visual world are then established.

In the second stage, the established visual representations are brought to mental processing, which is analogous to the feature binding stage (see the blue panel in Fig. 2). Only representations that are of propositional semantic features (in the form of a propositional statement, e.g., *this is a car*, and *the windows are broken*) enter this stage, therefore, all the mental representations are propositional in nature.

In the third stage, the newly established mental representations are assessed according to their relevance to the prototype (i.e., the extant mental representations that are established on the basis of prior experience and knowledge and are stored in long-term memory). The representations contained in the prototype are also propositional in nature (see the yellow panel in Fig. 2). For instance, if the prototype contains two mental representations, *cars have a neutral connotation* and *broken windows have a negative connotation*, then based on the relevance to the prototype, the two newly established mental representations in the example are evaluated differently. The representation *this is a car* is rendered as “neutral,” and the representation *the windows are broken* as “negative.” More specifically, this assessment process leads to two final propositional representations: *the fact that this is a car has a neutral connotation* and *the fact that the windows are broken has a negative connotation*. These two final mental representations are the output of this stage. It should be noted that individual differences might occur at this stage, because the prototype against which the newly established representations are evaluated is built upon prior knowledge and experience that might vary across individuals.

In the fourth stage, the final mental representations (the output of the assessment stage) are registered in working memory and transferred to implement cognitive-affective processes. In particular, both DD and ASD are quite likely to have representational differences in mind from health participants when presented with visual scenes of emotional and cognitive meanings. The representational differences guide the eye movements in the visual world, yielding eye movement patterns that reflect the final mental representations due to the computational processing of cognitive-affective information. For instance, DD patients have difficulty in disengaging from negative information^42^, and ASD patients have a low matching degree of proposition and prototype^43^. Therefore, reliable eye movement indicators (e.g., transit time, first fixation duration, total fixation duration, fixation proportions, and saccade path length) combined with AI algorithms, e.g., Naive Bayesian, logistic regression, Support Vector Machine (SVM), and deep learning, can serve as a reliable and accurate measure of cognitive-affective ToM.

**Fig. 3:**
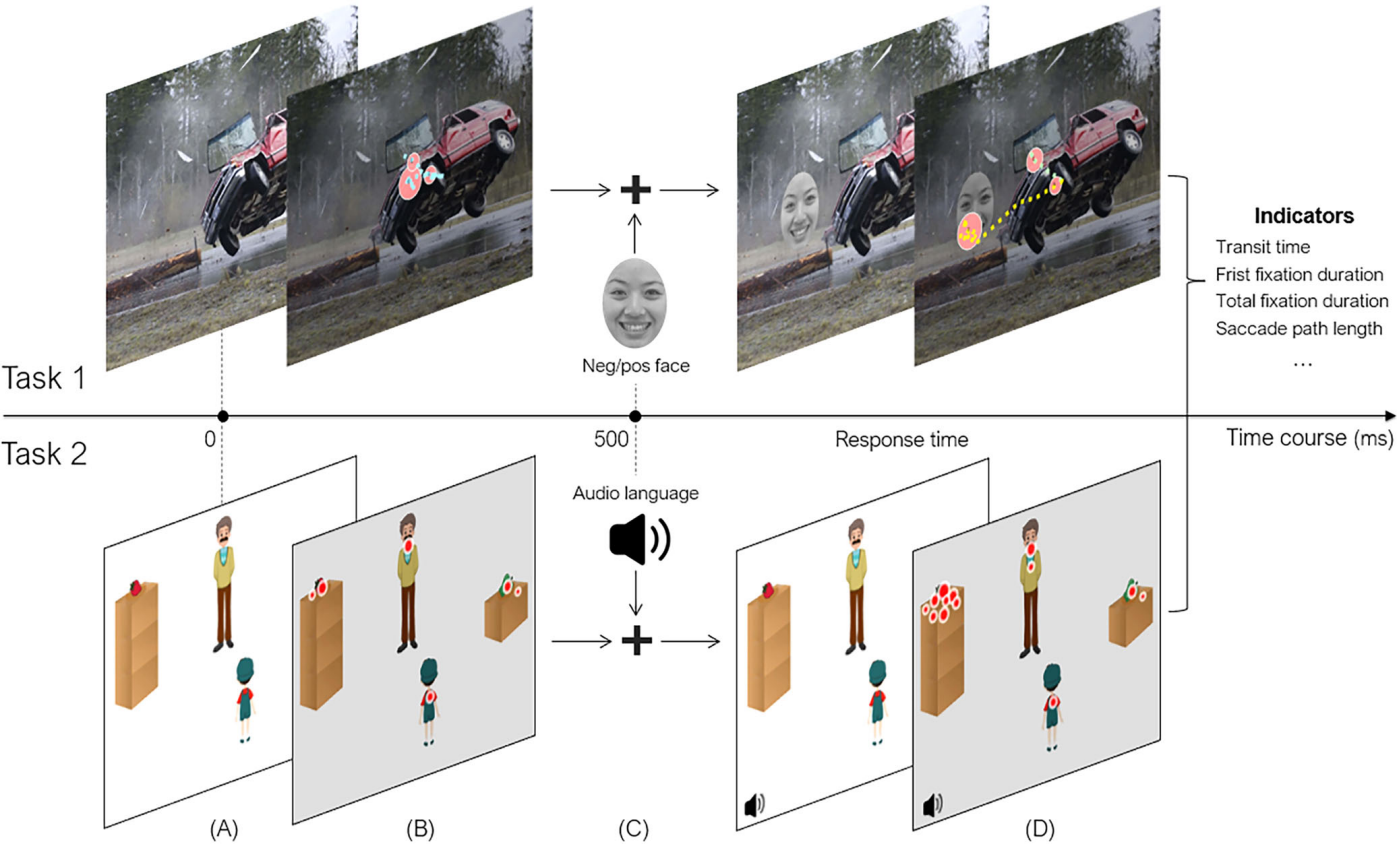
Audio-visual paradigm. Study 1: (**A**) A black background was shown to the participants for 1000 ms, and then a random positive or negative image was presented as the background scene. (**B**) Participants’ initial eye gaze patterns when the background scene was shown to them were recorded by a Tobii EyeX Controller eye tracker (sampling rate 60 Hz). (**C**) A random positive or negative face was presented randomly on the left or right of the background scene as the foreground stimulus in between 500 and 1000 ms. (**D**) The response time in which the participants judged the emotional attribute of the face was measured by pressing the button, and at the same time their eye movements were recorded. Study 2*:* (**A**) In the visual stimulus, a strawberry, the liked object by the boy character Kangkang, was placed on the left high box and a green pepper, the disliked object, was placed on the right low box. The social element that Kangkang is facing is either a man (+social) or a tree (−social). (**B**) Participants’ initial eye gaze patterns when the visual stimuli were first shown to them were recorded by an EyeLink 1000 plus eye tracker (sampling rate 500 Hz). (**C**) A spoken sentence saying, “Look, which object do you think Kangkang will reach for?” was presented 500 ms after the appearance of the visual stimulus. (**D**) Each participant’s eye movement was recorded from the onset of the verb for 1000 ms.

Our model is based on two tasks using the audio-visual paradigm with eye movement measurements (detailed descriptions of the paradigm are provided in Fig. 3). Study 1 was designed to investigate the difference in affective ToM between individuals with DD and healthy controls. Study 2 was designed to investigate the difference in cognitive ToM between typically developing children and children with ASD. Compared with traditional paradigms of ToM research, our paradigm, for the first time, innovatively combines the investigations of these two core components of ToM. In addition, our audio-visual paradigm is based on well-constructed visual scenes with emotional and cognitive meanings and large sample size (*n* > 700), and therefore the obtained indicators serve as a highly objective measure.

### Study 1: difference in affective ToM between individuals with DD and healthy controls

Two hundred and twelve people with diagnosed DD, including 79 males and 133 females, participated in the study (mean age = 32 (years);4 (months), SD = 12.79). Their diagnoses were confirmed by certified psychiatrists in hospitals using DSM-5 (APA, 2013), and were further complemented by two screening instruments, the Self-Rating Depression Scale (SDS) and the SCL-90 depression sub-scale^44,45^. In addition, 492 healthy controls without diagnosed DD, including 273 males and 219 females, participated as the control group (mean age = 34;1, SD = 14.24). The study was under the approval of the Ethics Committee of Beijing Anding Hospital, 201722FS-2, and written informed consent was obtained from all the participants. The participants were also recruited following the below eligibility criteria: (1) No history of previously diagnosed schizophrenia, schizophrenic affective disorder, or mental disorders associated with other diseases; (2) No history of alcohol or substance dependence; (3) Not on psychotropic medications for their conditions ; (4) Not suffering from any serious physical diseases that are not suitable to be included in this study. The experiments in this study were performed in accordance with the guidelines in the Declaration of Helsinki.

**Fig. 4:**
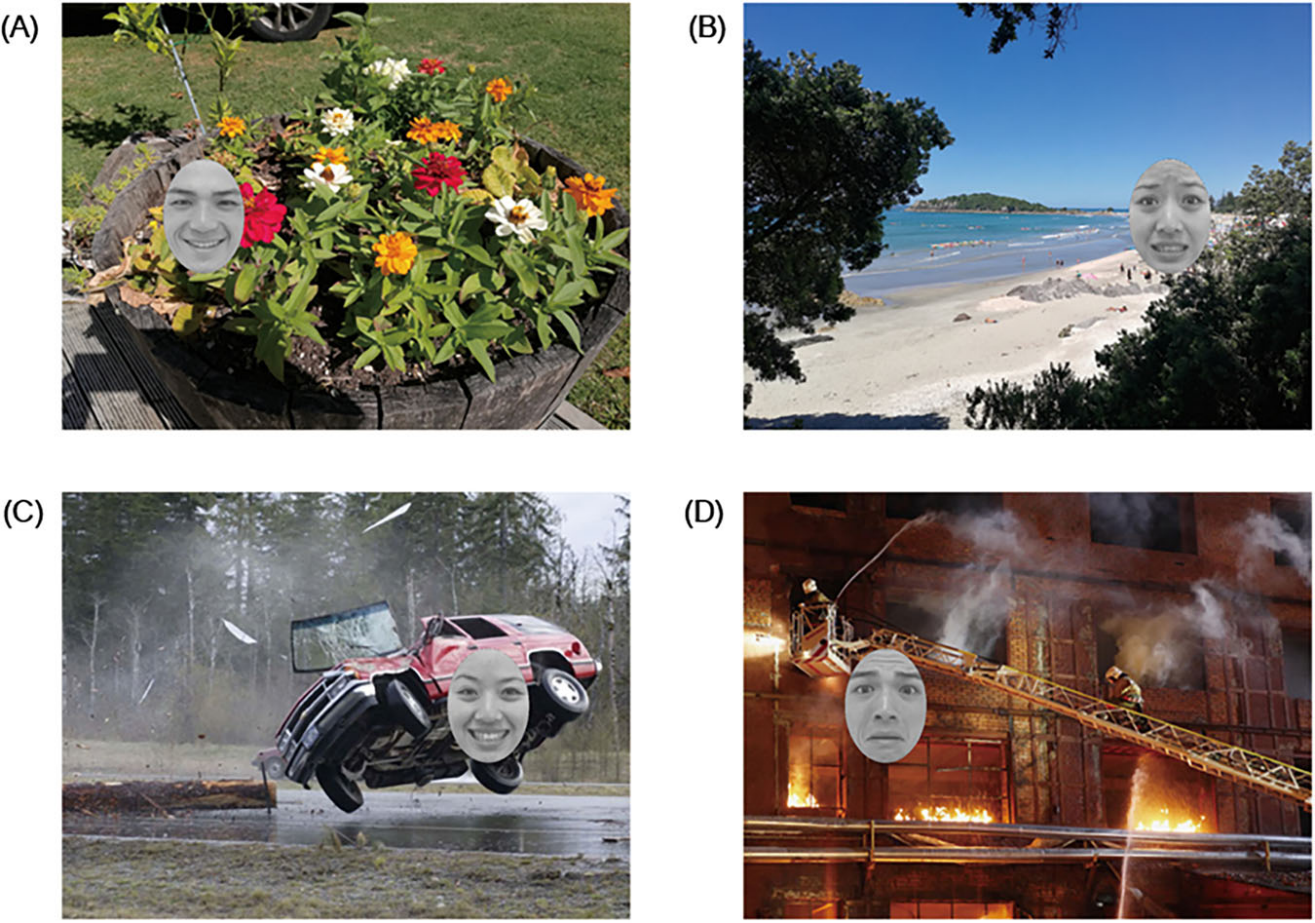
A typical visual stimulus of Study 1. Four combinations of the emotional status of the images and the faces: (**A**) positive image with positive face, (**B**) positive image with negative face, (**C**) negative image with positive face, and (**D**) negative image with negative face.

The experiment was conducted to investigate whether the attentional bias during emotion understanding could be used for DD identification. Using the competing-priming effect paradigm, emotional images and emotional faces were presented to the participants as visual stimuli. A total of four sets of visual stimuli were constructed. As shown in Fig. 4, the four sets varied in the combinations of the emotional status of the images and the faces: a positive image with a positive face, a positive image with a negative face, a negative image with a positive face, and a negative image with a negative face. 40 positive and 40 negative images were constructed as the background scenes using the ThuPIS database^46^. The emotional faces were selected from the Taiwanese Facial Expression Image Database^47^, including 8 positive and 8 negative faces serving as the foreground stimuli. Both are open access database. Across the 4 sets of visual stimuli, each set consisted of 20 trials made up of the image and the face with the corresponding emotional status. All trials in the lists were arranged in random order.

The experimental paradigm requires the participants to search for the presence of emotional faces in the background of emotional images, then judge the attributes of emotional faces. The reaction time data and eye movement data were recorded during this process. Before the actual test, each participant received a pre-test in which they needed to perform 8 consecutive trials correctly. The actual test consisted of 80 trials. Each trial proceeded as follows. First, a black background was shown to the participants for 1000 ms. Then, a random positive or negative image was presented as the background scene to attract the participants’ attention to the background. Following this, a random positive or negative face was presented randomly on the left or right of the background scene as the foreground stimulus in between 500 and 1000 ms. The participants were expected to switch their visual attention from the background scene to the foreground stimulus. Finally, the participants were asked to judge the emotional attribute of the faces by pressing the left (positive) or the right button (negative). Their response time was recorded at the same time. Response time indicates the speed at which the participant got distracted from the background scene and transferred to the foreground stimuli while judging the emotional status of the face. During the whole process, the participants’ eye movements were recorded using a Tobii EyeX Controller eye tracker with a sampling rate of 60 Hz. Prior to the formal experiment, participants went through a 4-point calibration procedure while seated approximately 60 cm from the screen. If the calibration error was beyond the threshold of 1° visual angle, the calibration and validation process repeated. The experimental stimuli were presented on a monitor with a resolution of 1600 × 900 pixels (21.5 inches) and programmed using C# with the framework of Windows Presentation Foundation.

This experimental paradigm fully integrates the idea of attentional bias hypothesis, mapping the effects of clinical depression onto attentional biases for emotional stimuli. During the experiment, participants were attracted by emotional images and emotional faces, and formed corresponding fixation points, which reflected the characteristics of attention orientation. In addition, to complete the attribute discrimination task of emotional faces, participants also needed to direct their gaze away from the emotional image area to the emotional face, further reflecting the ability to disengage attention. Finally, participants transferred their attention from the emotional image area to the emotional face area and made judgments, indicating attention transfer in the process of the experiment. By this means, the three attention components (attention orienting, attention disengagement, attention transfer) were unified in one paradigm. We believe that the current paradigm can accurately assess affective ToM, not the attentional control in the early visual stage, because the stimuli we devised were composed of not only facial images but also background images containing affective semantics. Although the facial images can be processed in early visual stage^48^, no strong evidence shows that the affective semantics of images can be processed in such an early stage. In addition, extant studies suggest that selective attention impairments in DD are specific to feature-based selective attention while spatial selective attention remains intact^49^. Therefore, by analyzing the eye movement trajectory information during the experiment, we can detect the differences of affective ToM between participants with DD and healthy controls.

### Study 2: difference in cognitive ToM between TD children and children with ASD

Three hundred and thirty-two Mandarin-speaking children with ASD, who were diagnosed using DSM-IV-TR (APA, 2000) and DSM-5 (APA, 2013) by hospitals, and reconfirmed by our research team using the Autism Diagnostic Observation Schedule (ADOS), participated in the study. All the participants with ASD met the autism cut-off of the ADOS, with a mean score of 10.5 and a standard deviation of 3.5. Six hundred and twelve age-matched TD Mandarin-speaking children participated as the control group. The participants’ verbal IQ scores were assessed using Wechsler Preschool and Primary Scale of IntelligenceTM-IV (CN). All the participants in the ASD and TD groups had verbal IQ scores above 85. The study was under the approval of the Ethics Committee of the School of Medicine, Tsinghua University, 20170018, and all the participants or their guardians provided written informed consent. The experiments in this study were performed in accordance with the guidelines in the Declaration of Helsinki.

**Fig. 5:**
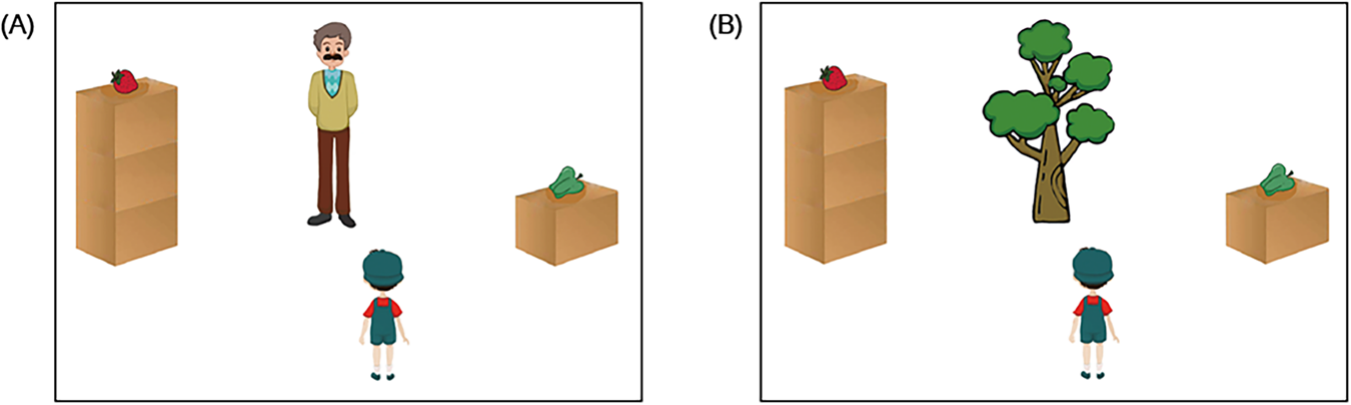
A typical visual stimulus of Study 2. (**A**) a visual stimulus with a man, and (**B**) a visual stimulus with a tree.

Sixteen pairs of visual stimuli were constructed by the research team. In each pair, two visual images were constructed, both containing a boy called Kangkang, two boxes (a high one and a low one) and two objects (one that Kangkang favors, referred to as the liked object, and the other one that he dislikes, referred to as the disliked object). The only difference between the two images was that in one of the pictures Kangkang was facing a man while in the other Kangkang was facing a tree. An example of the visual stimuli is presented in Fig. 5A, B. Across the trials, the audio stimulus that accompanied the visual images was always the same Mandarin spoken sentence: “Kan, ni juede Kangkang hui qu gou na yige?”[=Look, which object do you think Kangkang will reach for?]

In both Fig. 5A, B, a strawberry, the liked object of Kangkang, was placed on the top of the left high box, while a green pepper, the disliked object, was on the top of the right low box. The fact that the liked object was out of the reach of Kangkang made the different social meaning between the man in Fig. 5A and the tree in Fig. 5B critical—the man, rather than the tree, is both capable and ready to help. The experiment was conducted to investigate whether preschool Mandarin-speaking children with ASD were capable of making use of the social meaning of the man to predict Kangkang’s behavior (social cognition), as compared to TD children.

Across the 16 pairs of visual stimuli, both the position of the two boxes (i.e., whether they are on the left side or right side) and the location of the two objects (i.e., whether they are on the high box or low box) were counterbalanced. The 16 pairs of test objects were split into two lists, each list including only one of the test pictures in each pair (e.g., either Fig. 5A or Fig. 5B) accompanied by the same spoken sentence. Each list was made up of 8 trials with an old man (e.g., Fig. 5A) and 8 trials with a tree (e.g., Fig. 5B), and the number of trials in terms of which box was on which side and which object was on which box was equal: four trials with the liked object on the high box on the left, four trials with the liked object on the high box on the right, four trials with the liked object on the low box on the left, and four trials with the liked object on the low box on the right. All trials in the two lists were randomly arranged. Participants from experimental and control groups were assigned to one of the two experimental lists randomly. 166 5-year-old children with ASD, 150 TD 5-year-old and 156 TD 4-year-old children were presented with List 1, and 166 5-year-old children with ASD, 150 TD 5-year-old and 156 TD 4-year-old children were presented with List 2.

Each participant received two pre-tests and one actual test. The first pre-test was designed to create the contrast between the liked and disliked objects. Participants were presented with 16 pairs of liked and disliked objects that would be used in the test phase, and explicitly instructed by the experimenter about which were the liked objects of Kangkang, and which were the disliked objects. After the training period, they were tested using 12 pairs of objects to see whether they could discriminate between the liked and disliked ones. Only children who passed all the test trials moved onto the next phase. The second phase was to establish the awareness of height. Participants were told that the old man could reach the high box while Kangkang could only reach the low box. After that, the participants were shown 12 test pictures, half of which contained a liked object on the high box and another half of which contained a disliked object on the high box. They were asked to indicate by pointing to the object that Kangkang could or could not reach for. Only children who answered all 12 questions correctly proceeded to the actual test phase.

The final test phase was to investigate whether participants could use the social cues embedded in the visual scenes to make inferences about others’ minds and behavior. Sixteen visual images were displayed by a monitor together with a spoken sentence saying “Look, which object do you think Kangkang will reach for?”, which was presented 500 ms after the visual stimulus appeared. Participants were seated approximately 64 cm from a 21 inch, 4:3 color monitor with 1024 × 768 pixel resolution. They were not asked to give any explicit responses, but just viewed the visual scenes freely. An EyeLink 1000 plus eye tracker was used to record the participants’ eye movements from the onset of the verb “reach” for 1000 ms because we believe they could infer Kangkang’s behavior after hearing the verb. The eye tracker was run under the free-to-move head mode with the monocular sampling rate of 500 Hz and a spatial resolution of 0.01 and an average error of less than 0.5.

The critical manipulation here was the presence of an old man or a tree when the liked object was placed on the high box. Note that typically developing children as young as 24 months of age know that the presence of a man indicates that the man could help them reach an object (e.g., refs. ^50,51^). If participants could make social inferences, they would demonstrate different eye movement patterns. To be more specific, they should show more fixations to the liked objects on the high box when an old man rather than when a tree was in the visual scene, because they inferred that the man could reach for the high box and he was willing to offer help while the tree could not, and they also inferred that Kangkang believed that the old man could help him achieve his goal. Based on these inferences, they would further reason about Kangkang’s action: he was likely to reach for the liked object placed on the high box when the old man was present, but he probably would reach for the disliked object placed on the low box when a tree was present. Therefore, there should be a significant difference in the eye gaze patterns between these two critical settings, by which we can further understand whether children could make inferences based on social cues in an established visual world. Note that no difference in eye movements was expected to exist when the liked object was placed on the low box, since Kangkang could reach the object by himself.

### Reporting summary

Further information on research design is available in the Nature Research Reporting Summary linked to this article.

## Results

**Fig. 6:**
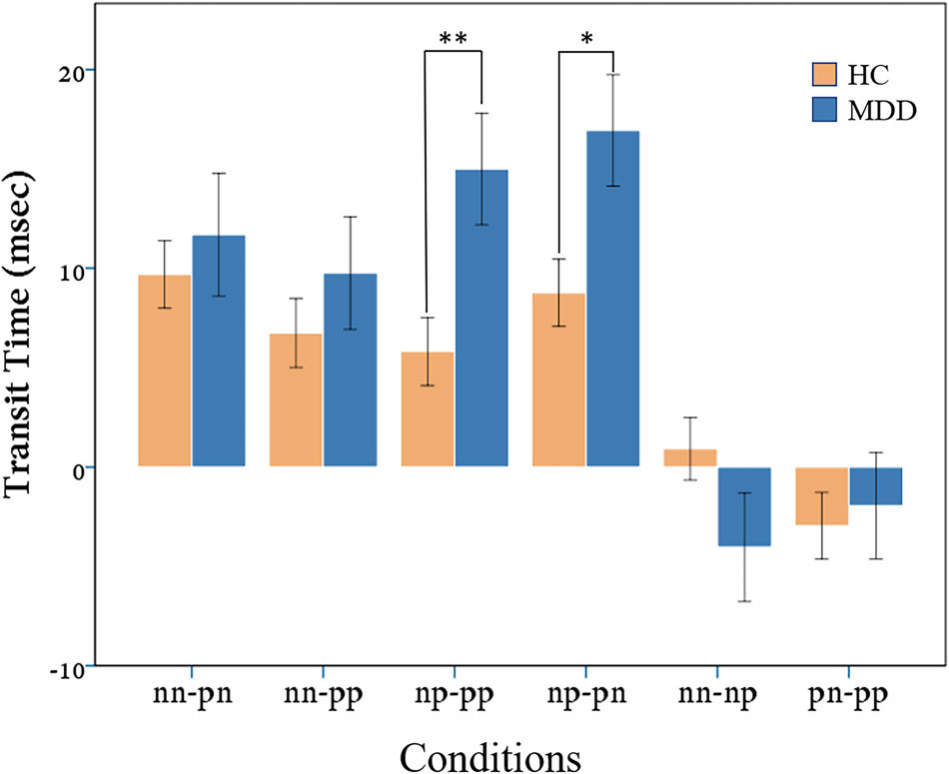
Results of Study 1 using the audio-visual paradigm. The x-axis contains 6 conditions of difference in transit time between different foreground and background combinations (e.g. np-pp, where np and pp are two combinations, np indicates negative background with positive face and pp indicates positive background with positive face. The difference in transit time was then calculated as the transit time from negative background to positive face minus the transit time from positive background to positive face. Other conditions can be interpreted accordingly.). (error bars indicate standard errors, asterisk (*) denotes *p* < 0.05).

**Fig. 7:**
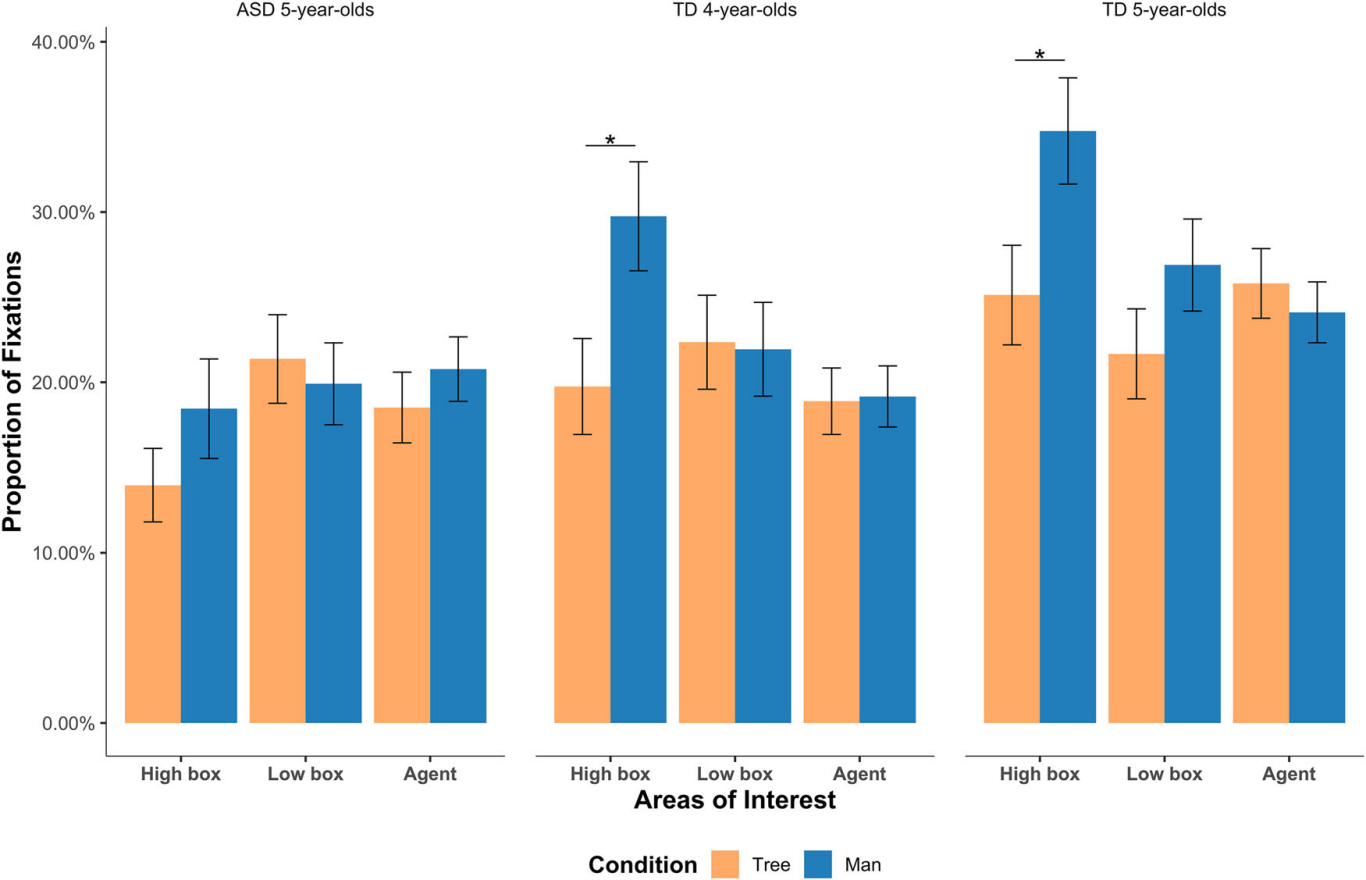
Results of Study 2 using the audio-visual paradigm. Fixation proportions of the ASD 5-year-olds, the TD 4-year-olds and the TD 5-year-olds on the high box with the liked item (the left panel), on the low box with the liked item (the middle panel) and on the agent (the right panel) in scenes where there was a man vs. in scenes where there was a tree. (error bars indicate standard errors, asterisk (*) denotes *p* < 0.05).

The results are illustrated in Figs. 6 and 7. Error bars indicate standard errors. Figure 6 displays the results of Study 1. Eye movements were recorded in Study 1 which reflect the detailed attention deployment information (e.g., orientation, release, transfer) about how the participant got distracted from the background scene to the foreground stimulus. We followed the standard procedure for pre-processing eye movement data. First, the participants whose eye fixation durations were less than 80 ms (the minimum time to form a fixation) on more than 10 trials were excluded from further analysis, because fixation durations less than 80 ms indicate meaningless eye movements due to technical issues or lack of cooperation from the participants (*N* = 68). Second, participants whose average error rate was more than 20% either in the entire experiment or in one condition were excluded (*N* = 37). In addition, the data of participants with values greater than 3 SD above the average were also excluded as outliers (*N* = 76). This procedure was implemented to ensure that only meaningful eye movements were included in the final analyses. The procedure led to the inclusion of 523 participants in the final analyses. According to the attentional bias hypothesis, participants with DD should exhibit more difficulties than healthy controls when disengaging attention from negative stimuli and then transferring their attention to positive stimuli. And this can be characterized as transit time which is defined as the time difference between the onset of the positive/negative face and the time when the first fixation point formed on the face. Therefore, we calculated the difference in transit time between any two of the four foreground and background combinations. This process resulted in six combinations: nn-pn, nn-pp, np-pp, np-pn, nn-pn, and pn-pp. For further clarification, consider np-pp as an example, where np and pp are two combinations, np indicates negative (n) background with positive (p) face and pp indicates positive (p) background with positive face (p). The difference of transit time was then calculated as the transit time from negative background to positive face minus the transit time from positive background to positive face (np-pp). Other conditions can be interpreted accordingly. Therefore, np-pp represents the transit time difference between negative background with positive face and positive background with positive face. A significant positive value of this condition would indicate the difficulty in disengaging attention from negative stimuli. To test the effect of participant group on transit time differences, one-way multivariate analysis of variance of participant groups was performed with effect size calculated with partial eta squared (using IBM SPSS Statistics 24), and the result shows significant difference between the two groups (Wilks’ Lambda = 3.498, *p* < 0.01, ${\eta }_{p}^{2}$ = 0.136). To further investigate this significant difference, Fig. 6 shows the transit time difference for the two groups in each condition. As shown in the figure, only np-pp (*F*(1,522) = 7.87, *p* < 0.01, ${\eta }_{p}^{2}$ = 0.014) and np-pn (*F*(1,522) = 6.35, *p* < 0.05, $\,{\eta }_{p}^{2}$ = 0.011), but no other conditions (all *p*s > 0.11), show significantly larger transit time differences between the DD group and the healthy controls, indicating that it was more challenging for participants with DD as compared with healthy controls in disengaging attention from negative stimuli and reducing preference to positive stimuli. Note that both the DD group and the healthy controls displayed a positive value of transit time difference, suggesting that both groups took longer time to disengage attention from negative stimuli than from positive ones. However, the magnitude of this difference was significantly greater for the DD group than for the healthy controls, indicating a second-order difference. This second-order difference might imply a fundamental disparity between the DD group and the healthy controls when processing emotional stimuli. Let’s discuss the non-significant conditions in more detail. The nn-pp condition occurred when both the background and the facial stimuli were negative for nn, but positive for pp, in which there was no competition between the positive and negative stimuli for attention, and thus failing to demonstrate a greater difficulty in disengaging attention from negative stimuli. Similarly, the lack of disengagement also applies to the nn-pn, nn-np, and pn-pp conditions, where there was no disengagement of attention from the negative background to the positive facial stimuli. To explain the non-significant results of the nn-np and pn-pp conditions, we present the following speculations. The nn-np value for the DD group was negative, indicating that they were less likely to transit their attention towards the positive stimuli when the faces were positive, thus yielding longer attention shift time for the np condition than for the nn condition. Concerning the pn-pp condition, the difference value was greater in the healthy controls than in the DD group, presumably because compared with the participants with DD, the healthy controls were less likely to shift their attention towards the negative stimuli in the pn condition, leading to a larger difference in the pn-pp condition. Overall, these findings verified the attentional bias hypothesis of people with DD by showing that they exhibited significant difficulties in disengaging attention from negative stimuli and reducing attention to positive stimuli, and thereby serving as a sensitive measure of individuals’ affective ToM.

Figure 7 provides the results of Study 2. Eye movement data of all trials was included for both ASD and TD children according to our inclusion criteria. There are four areas of interest in the visual image: the high box area, the low box area, the social element area (man or tree), and the area including Kangkang. Each participant’s proportion of fixations onto these four areas per trial as the dependent variable was computed and further analyzed using generalized linear mixed models (GLMMs). GLMMs were computed for each participant group that included condition (i.e., when an old man versus when a tree was in the visual scene) as the fixed effect, and trial and participant as two random terms. The statistical models were chosen, because the eye fixation patterns were non-parametric binomial data in nature, and this model could include both fixed effects and by-participant as well as by-trial random effects. The model fitting process was realized using the *glmer* function in the lme4 package (v1.1.19) (Bates et al. 2013) under the R (v3.5.2) software environment (R Development Core Team 2017).

As indicated in Fig. 7, when the liked object was placed on the high box, 4-year-old and 5-year-old TD children showed more fixations on the high box when the man was present compared to when the tree was present. A significant difference between the two critical settings was observed for both the TD 4-year-old children (*b* = 1.21, *z* = 3.45, *p* < 0.01) and the TD 5-year-old children (*b* = 0.14, *z* = 3.18, *p* < 0.01). However, this effect was not found for the 5-year-old ASD group (*b* = 0.77, *z* = 1.16, *p* = 0.35). In addition, there was no difference in the fixation proportions when the man was in the visual scene vs. when the tree was in the visual scene under the condition in which the liked object was placed on the low box. The results clearly indicate a difference in social inference between the TD children and children with ASD, thus serving as a sensitive measure of children’s cognitive ToM.

## Discussions

The findings of the two studies clearly show how individuals with DD and ASD differ from healthy controls in cognitive-affective processes, and the obtained eye movement indicators are reliable and effective measures of cognitive-affective ToM. In addition, the findings can also inform us about the applicability and generalizability of our model. We discuss how V-ToM accounts for typical and atypical cognitive-affective ToM in relation to the findings of Study1 and Study 2 respectively. In particular, we focus on how the identification of DD and ASD can be related to the four stages proposed in V-ToM.

**Fig. 8:**
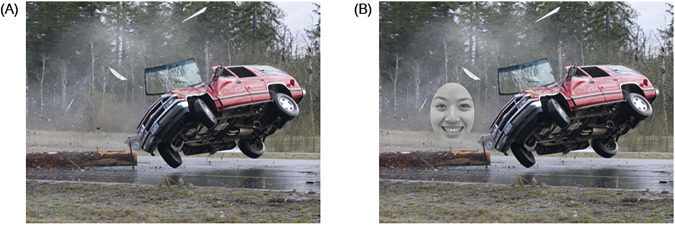
A example visual image of Study 1. (**A**) a car accident was first presented, and (**B**) an emotional face stimulus was then shown in the image.

In Study 1, when presented with a typical visual stimulus like Fig. 8, in which a car was moving on the road and then an accident happened, the participants first visually processed the scene, by means of segmenting the entire car from the background, constructing the border of the car, determining the orientation of the car, and analyzing the color of the car. Representations of the visual scene were then established (S1. Visual processing).

After the visual representations were established, mental processing commenced immediately. At this stage, the mental representations were stored in working memory in the form of propositional statements, such as *this is a car, the car is dented, the windows are broken, the car tilted and flew off the ground, the car is red*, and so on (S2. Mental processing).

Then, the participants compared the mental representations established in S2 with the cognitive prototype stored in long-term memory, and established relevant prototypical representations in the form of propositions as well. Relevant prototypical representations included *cars have a neutral connotation, dented surface has a negative connotation, broken windows have a negative connotation, tilted and flew-off conditions have a negative connotation, red color has a neutral connotation*. Based on the prototype, the assessment process leads to corresponding final propositional representations: *the fact that this is a car has a neutral connotation*, *the fact that the car is dented has a negative connotation*, *the fact that the windows are broken has a negative connotation*, *the fact that the car tilted and flew off the ground has a negative connotation* and *the fact that the car is red has a neutral connotation*. These relevant mental presentations are the output of this stage (S3. Assessment).

The final representations computed in S3 were then registered in working memory and guided the eye movements in the visual world. In the task, the participants were then presented with a positive or negative face image, and were asked to shift attention from the background image and make judgments on the attribute of the face. Individuals with DD exhibited different eye movement patterns than healthy controls, presumably due to the differences between the two groups in any of the four stages proposed in our model. For example, individuals with DD might process visual information differently than healthy controls, leading to different visual representations (S1); they might establish mental representations that differ from healthy controls in degrees of positivity or negativity (S2); they might have different emotionally relevant prototypes from those of healthy controls (S3), or they might have difficulty in disengaging from negative information (S4). Differences in any of the four stages would lead to different eye movement patterns by individuals with DD as compared with healthy controls. Therefore, the eye movement patterns obtained from Study 1, e.g., start time of transfer (from face appeared to attention shifted) and transfer speed (from background scene to foreground stimuli), can serve as reliable indicators of emotion understanding (S4. Transfer and implementation).

In Study 2, when presented with a typical visual stimulus like Fig. 5, where the liked object was placed on the high box and an old man (Fig. 5A) or a tree (Fig. 5B) was present in the scene, the participants first visually processed the images, by means of segmenting and segregating the elements from the background, and detecting the basic features of the elements, including border, contour, color, etc. Representations of the visual image were then established (S1. Visual processing).

Mental processing commenced immediately after S1. At this stage, the mental representations are established and stored in the form of propositional semantic statements. For instance, the mental representations of Fig. 5A include, *this is Kangkang*, *Kangkang is short*, *this is a man, the man is tall*, *this is a high box*, *this is a strawberry*, *the strawberry is the liked object of Kangkang*, *the strawberry is outside Kangkang’s reach*, *the man can reach the strawberry*, *this is a low box*, *this is a green pepper*, *the green pepper is the disliked object of Kangkang*, *Kangkang can reach the green pepper*, *the man can reach the green pepper*, and so on (S2. Mental processing).

The mental representations established in S2 are assessed according to their relevance to the prototype. The prototype is also represented in the form of propositional statements and is stored in long-term memory. In the social setting of Fig. 5A, the two relevant prototypical representations are: *men are highly social beings and are willing to help* and *boys believe that men are willing to help*. Evaluation of the established representations against the prototypical representations leads to two new mental representations: *the man is ready to help and is going to help* and *Kangkang believes that the man is ready to help and is going to help*. By contrast, in the social setting of Fig. 5B, the two relevant prototypical representations are: *trees are not social beings and are unable to help* and *boys believe that trees are unable to help*. The assessment against the prototype leads to two new mental representations: *the tree is unable to help* and *Kangkang believes that the tree is unable to help* (S3. Assessment).

The two new mental representations computed in S3 are registered in working memory and are transferred to guide the eye movements in the visual world. In this task, the participants are going to predict Kangkang’s behavior based on the relevant mental representations and make the social inference that Kangkang could ask the man to help him reach the liked object placed on the high box when presented with Fig. 5A. In contrast, when shown Fig. 5B, the participants would infer that Kangkang could not ask the tree for help and could only reach for the disliked object placed on the low box. Thus, the participants should show more looks at the area of the high box in the social setting of Fig. 5A containing the man, as compared to the same area of the high box in Fig. 5B with the tree (S4. Transfer and implementation).

When presented with visual images like Fig. 5, children with ASD exhibited different eye movement patterns than their TD peers, presumably due to the difference between the two groups in any of the four stages described in our model. For instance, children with ASD might exhibit certain kinds of preference in processing and integrating visual information, thus leading to different visual representations than those of TD children (S1); they might establish different mental representations from those of their TD peers (S2); they might have a different set of socially relevant prototypical representations as compared with TD children (S3); or they might have a low matching degree of proposition and prototype (S4). Differences in any of the four stages would lead to different eye movement patterns by children with ASD as compared with TD children. Thus, the obtained eye movement patterns in Study 2 can be used as reliable indicators of social cognition. An anonymous reviewer raised an interesting question: whether there is an emotional development factor that could explain the eye movement pattern in Study 2. More specifically, the reviewer asked whether it is possible that ASD children do not develop emotionally at the same rate as TD children. We agree that this question is worthy of further exploration, but we will leave that for future research that directly investigates the connection between emotional development and theory of mind/social cognition in both ASD and TD children. Nonetheless, this potential factor does not affect our findings attesting to the significant difference between ASD and TD children.

The present paper provides a fine-grained conceptual framework of cognitive-affective ToM by dividing the relevant underlying processes into four stages. Compared with traditional models, our model, for the first time, innovatively integrates the two core components of ToM, the cognitive and affective components, into the construction of ToM. In addition, by detailed analysis of the four cognitive stages, our model allows us to explain how humans process cognitive-affective information in a fine-grained manner. Each stage is measurable, thus enabling us to make inferences about potential difficulties that might occur in each of the four stages. More specifically, we propose a theory of V-ToM by devising a novel audio-visual paradigm and by constructing visual scenes with cognitive and emotional meanings. In addition, the audio-visual paradigm is based on large sample size (*n* > 700) and thus the obtained eye movement patterns are robust and reliable indicators of the underlying cognitive-affective processes. The findings also suggest that the obtained eye movement patterns can be used effectively to distinguish between typical and atypical populations in the two core domains of ToM, and thus can potentially serve as clinical markers for DD and ASD.

We also wish to point out two limitations of the current study. The first limitation comes with the limited age range of the DD and ASD participants. We recruited only child participants for the cognitive ToM study and only adult participants for the affective ToM study. Ideally both adults and children should be included for each study, so that potential confounds due to age difference could be controlled for. The second limitation concerns the limited disorders being investigated in the study. The current study focused on the DD and ASD populations without comparing them to other DSM disorders. Ideally, to provide stronger evidence for specificity, future research should investigate a broader array of individuals with DSM disorders using the same paradigm, and then compare those groups against one another both within the adult population and the child/adolescent population.

To conclude, future work is needed to address the “real world” implications of these deficits and to develop effective transdiagnostic interventions for those individuals that are adversely affected. Traditional diagnostic methods for DD and ASD are largely based on the subjective assessment of the clinicians. By contrast, our model features a more objective and efficient measurement of DD and ASD. By extracting participants’ eye movement indicators from multiple dimensions automatically, we are able to identify the potential sources of their difficulties, thereby leading to more precise and reliable identification and intervention programs for individuals with DD and those with ASD. Note that the computability of the proposed V-ToM model operationalized abstract concepts of ToM into measurable processes that are potentially to be interfaced with AI technology, leading to AI-aided precision identification of mental disorders based on digital medicine.

### Supplementary information

Reporting Summary

## Data Availability

The full set of data and materials that support the findings of the experiments are available from the Beijing TeeView Technology Co., Ltd. Restrictions apply to the availability of these data, which were used under license for the experiments. Data are available on the request from the corresponding author H.M. with the permission of Beijing TeeView Technology Co., Ltd.

## Code availability

The data were analyzed using standard statistical packages as discussed in the text. Analysis code is available on the request from the corresponding author H.M. with the permission of Beijing TeeView Technology Co., Ltd.
