# Supplementary material for: A conceptual framework of cognitive-affective theory of mind: towards a precision identification of mental disorders
Source: Npj Ment Health Res. 2023 Aug 10;2:12. doi: 10.1038/s44184-023-00031-0 (PMC10955940; doi:10.1038/s44184-023-00031-0)
Supplement: Supplementary file 1 — Reporting Summary [file 44184_2023_31_MOESM1_ESM.pdf]

nature portfolio

Corresponding author(s): Huimin Ma

Last updated by author(s): 2023/05/10

Reporting Summary

Nature Portfolio wishes to improve the reproducibility of the work that we publish. This form provides structure for consistency and transparency in reporting. For further information on Nature Portfolio policies, see our Editorial Policies and the Editorial Policy Checklist.

Statistics

For all statistical analyses, confirm that the following items are present in the figure legend, table legend, main text, or Methods section.

n/a	Confirmed	
☐	☒	The exact sample size (*n*) for each experimental group/condition, given as a discrete number and unit of measurement
☒	☐	A statement on whether measurements were taken from distinct samples or whether the same sample was measured repeatedly
☐	☒	The statistical test(s) used AND whether they are one- or two-sided<br>*Only common tests should be described solely by name; describe more complex techniques in the Methods section.*
☒	☐	A description of all covariates tested
☐	☒	A description of any assumptions or corrections, such as tests of normality and adjustment for multiple comparisons
☐	☒	A full description of the statistical parameters including central tendency (e.g. means) or other basic estimates (e.g. regression coefficient) AND variation (e.g. standard deviation) or associated estimates of uncertainty (e.g. confidence intervals)
☐	☒	For null hypothesis testing, the test statistic (e.g. *F*, *t*, *r*) with confidence intervals, effect sizes, degrees of freedom and *P* value noted<br>*Give P values as exact values whenever suitable.*
☒	☐	For Bayesian analysis, information on the choice of priors and Markov chain Monte Carlo settings
☒	☐	For hierarchical and complex designs, identification of the appropriate level for tests and full reporting of outcomes
☐	☒	Estimates of effect sizes (e.g. Cohen's *d*, Pearson's *r*), indicating how they were calculated

Our web collection on statistics for biologists contains articles on many of the points above.

Software and code

Policy information about availability of computer code

Data collection	The participants' eye movements were recorded using a Tobii EyeX eye tracker in Study 1 and an EyeLink 1000 plus eye tracker in Study 2.
Data analysis	The multivariate analysis of variance was performed with IBM SPSS Statistics 24. The model fitting process was realized using the glmer function in the lme4 package (v1.1.19) (Bates et al. 2013) under the R (v3.5.2) software environment (R Development Core Team 2017).

For manuscripts utilizing custom algorithms or software that are central to the research but not yet described in published literature, software must be made available to editors and reviewers. We strongly encourage code deposition in a community repository (e.g. GitHub). See the Nature Portfolio guidelines for submitting code & software for further information.

Data

Policy information about availability of data

All manuscripts must include a data availability statement. This statement should provide the following information, where applicable:

- Accession codes, unique identifiers, or web links for publicly available datasets
- A description of any restrictions on data availability
- For clinical datasets or third party data, please ensure that the statement adheres to our policy

The full set of data and materials that support the findings of the experiments are available from the Beijing TeeView Technology Co., Ltd. Restrictions apply to the availability of these data, which were used under license for the experiments. Data are available on the request from the corresponding author H.M. with the permission of Beijing TeeView Technology Co., Ltd.

Research involving human participants, their data, or biological material

Policy information about studies with human participants or human data. See also policy information about sex, gender (identity/presentation), and sexual orientation and race, ethnicity and racism.

Reporting on sex and gender	In Study 1, two hundred and twelve people with diagnosed DD, including 79 males and 133 females, participated in the study (mean age = 32(years);4(months), SD = 12.79). In addition, 492 healthy controls without diagnosed DD, including 273 males and 219 females, participated as the control group (mean age = 34;1, SD =14.24). In Study 2, three hundred and thirty-two Mandarin-speaking children with ASD, who were diagnosed using DSM-IV-TR (APA, 2000) and DSM-5 (APA, 2013) by hospitals, and reconfirmed by our research team using the Autism Diagnostic Observation Schedule (ADOS), participated in the study.612 age-matched TD Mandarin-speaking children participated as the control group.
Reporting on race, ethnicity, or other socially relevant groupings	N/A
Population characteristics	Healthy controls, participants with DD, and participants with ASD
Recruitment	The participants in study 1 were recruited following the below eligibility criteria: 1) No history of previously diagnosed schizophrenia, schizophrenic affective disorder, or mental disorders associated with other diseases; 2) No history of alcohol or substance dependence;3) Not on psychotropic medications for their conditions ; 4) Not suffering from any serious physical diseases that are not suitable to be included in this study. The participants in Study 2 were Mandarin-speaking children with ASD, who were diagnosed using DSM-IV-TR (APA, 2000) and DSM-5 (APA, 2013) by hospitals, and reconfirmed by our research team using the Autism Diagnostic Observation Schedule (ADOS). The experiments in the two studies were performed in accordance with the guidelines in the Declaration of Helsinki.
Ethics oversight	The study was under the approval of the Ethics Committee of Beijing Anding Hospital, 201722FS-2, and the Ethics Committee of the School of Medicine, Tsinghua University, 20170018.

Note that full information on the approval of the study protocol must also be provided in the manuscript.

Field-specific reporting

Please select the one below that is the best fit for your research. If you are not sure, read the appropriate sections before making your selection.

☐ Life sciences ☒ Behavioural & social sciences ☐ Ecological, evolutionary & environmental sciences

For a reference copy of the document with all sections, see nature.com/documents/nr-reporting-summary-flat.pdf

Behavioural & social sciences study design

All studies must disclose on these points even when the disclosure is negative.

Study description	The studies are both quantitative cross-sectional studies.
Research sample	In Study 1, two hundred and twelve people with diagnosed DD, including 79 males and 133 females, participated in the study (mean age = 32(years);4(months), SD = 12.79). In addition, 492 healthy controls without diagnosed DD, including 273 males and 219 females, participated as the control group (mean age = 34;1, SD =14.24).<br>In Study 2, three hundred and thirty-two Mandarin-speaking children with ASD participated in the study, 612 age-matched TD Mandarin-speaking children participated as the control group.
Sampling strategy	The participants in study 1 were recruited following the below eligibility criteria: 1) No history of previously diagnosed schizophrenia, schizophrenic affective disorder, or mental disorders associated with other diseases; 2) No history of alcohol or substance dependence;3) Not on psychotropic medications for their conditions ; 4) Not suffering from any serious physical diseases that are not suitable to be included in this study. The participants in Study 2 were Mandarin-speaking children with ASD, who were diagnosed using DSM-IV-TR (APA, 2000) and DSM-5 (APA, 2013) by hospitals, and reconfirmed by our research team using the Autism Diagnostic Observation Schedule (ADOS). The experiments in the two studies were performed in accordance with the guidelines in the Declaration of Helsinki.
Data collection	The participants' eye movements were recorded using a Tobii EyeX eye tracker in Study 1 and an EyeLink 1000 plus eye tracker in Study 2. The participants were blind to the study hypothesis during data collection.
Timing	Data collection are from Oct. 2017 to Nov. 2018 for Study 1, and from Mar. 2017 to Mar. 2019 for Study 2.
Data exclusions	In Study 1, we followed the standard procedure for pre-processing eye movement data. First, the participants whose eye fixation durations were less than 80ms (the minimum time to form a fixation) on more than 10 trials were excluded from further analysis, because fixation durations less than 80ms indicate meaningless eye movements due tothis suggests that the recording of eye movement data may have been incomplete, possibly due to technical issues or lack of cooperation from the participantssubjects . (N=68). MoreoverSecond, participants whose average error rate was more than 20% either in the entire experiment or in one condition were excluded (N=37). In addition, the data of participants with values greater than 3SD above the average were also

	excluded as outliers (N=76). This procedure was implemented to ensure that only meaningful eye movements were included in the final analyses. In Study 2, eye movement data of all trials was included for both ASD and TD children according to our inclusion criteria.
Non-participation	Not applicable
Randomization	Not applicable

Reporting for specific materials, systems and methods

We require information from authors about some types of materials, experimental systems and methods used in many studies. Here, indicate whether each material, system or method listed is relevant to your study. If you are not sure if a list item applies to your research, read the appropriate section before selecting a response.

Materials & experimental systems

n/a	Involved in the study
☒	☐ Antibodies
☒	☐ Eukaryotic cell lines
☒	☐ Palaeontology and archaeology
☒	☐ Animals and other organisms
☒	☐ Clinical data
☒	☐ Dual use research of concern
☒	☐ Plants

Methods

n/a	Involved in the study
☒	☐ ChIP-seq
☒	☐ Flow cytometry
☒	☐ MRI-based neuroimaging
